# Reporter Mice for Gene Editing: A Key Tool for Advancing Gene Therapy of Rare Diseases

**DOI:** 10.3390/cells13171508

**Published:** 2024-09-09

**Authors:** Siang Li, Cord Brakebusch

**Affiliations:** Biotech Research and Innovation Center (BRIC), University of Copenhagen, Ole Maaløes vej 5, 2200 Copenhagen, Denmark; siang.li@bric.ku.dk

**Keywords:** gene editing, reporter mice, CRISPR, rare diseases

## Abstract

Most rare diseases are caused by mutations and can have devastating consequences. Precise gene editing by CRISPR/Cas is an exciting possibility for helping these patients, if no irreversible developmental defects have occurred. To optimize gene editing therapy, reporter mice for gene editing have been generated which, by expression of reporter genes, indicate the efficiency of precise and imprecise gene editing. These mice are important tools for testing and comparing novel gene editing methodologies. This review provides a comprehensive overview of reporter mice for gene editing which all have been used for monitoring CRISPR/Cas-mediated gene editing involving DNA double-strand breaks (DSBs). Furthermore, we discuss how reporter mice can be used for quickly checking genetic alterations by base editing (BE) or prime editing (PE).

## 1. Introduction

In recent decades, there has been remarkable progress in our comprehension of human genetics, largely propelled by advances in genome editing technologies. While genome sequencing was used to identify candidate mutations underlying rare diseases, the introduction of these mutations into model systems by gene editing technologies validated their functional relevance and allowed us to study their biological consequences in detail. Mice with a constitutive or tissue-specific knock-out of a gene can model rare diseases caused by a loss-of-function mutation. Introducing the patient mutation into mice by gene editing (knock-in mice) will prove the biological role of the mutation and is crucial for rare diseases induced by gain-of-function mutations. Moreover, gene editing also opened new avenues for potentially curing rare diseases caused by genetic mutations.

There are two primary approaches to gene editing therapy: in vivo and ex vivo [1,2]. In vivo therapy involves delivering the therapeutic genome editing elements directly to the patient. The in vivo therapy approach relies on the high efficiency of gene correction in the target cells, which could be stem cells but also non-dividing cells such as neurons or cardiomyocytes. Adverse effects of the gene editing tools or the transfer methodology might occur. Ex vivo therapy, on the other hand, involves genetically modifying cells or tissues outside the patient’s body before reintroducing them back to the host [1]. During in vitro culture, an enrichment of successfully repaired stem cells may be possible.

**Figure 1:**
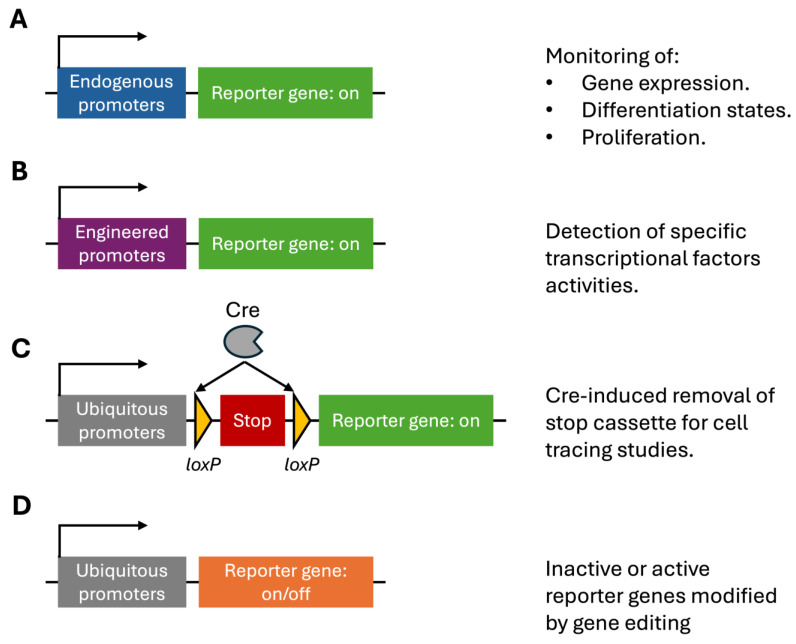
Overview of the reporter mice applications. (**A**–**D**) Different reporter mouse types are presented. On the left side the genetic elements are presented and on the right side the application.

**Figure 2:**
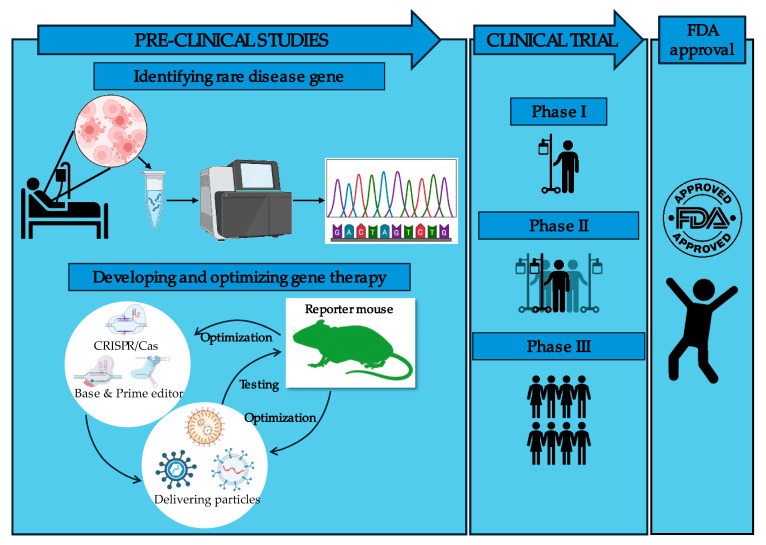
Overview of the usage of gene editing reporter mice in gene therapy research. Reporter mice are used in preclinical studies to test and optimize the gene editing procedure before advancing to clinical trials.

For both in vivo and ex vivo therapy it would be excellent to have a simple animal model to test the efficiency of different methodological approaches and to optimize their efficiency. This is the purpose of gene editing reporter mice, which monitor the success of gene editing by expression of easily detectable markers (Figure 1). Besides gene editing, reporter mice have been used to monitor the activity of endogenous promoters to check for differentiation status, proliferation, or the expression of a specific gene. Reporters coupled to engineered promoters have been used to test promoter-targeting drugs. Finally, induced permanent switch-on of a reporter was used to track specific cells and their fate in vivo. Such gene editing reporter mice can play an important role in the preclinical development and optimization of gene therapies for rare diseases (Figure 2).

For in vivo gene editing, different delivery methods could be tested, such as viral vectors, lipid nanoparticles, or exosomes with the efficiency of gene editing and the identity of targeted cells as major readouts [3]. Additionally, adverse effects including immune responses could be studied. For ex vivo therapy, the efficiency of in vitro gene editing will be determined in primary cells derived from reporter mice. In addition, gene-edited cells can be tracked after transplantation by their reporter.

## 2. Gene Editing Techniques

Several different techniques have been developed for gene editing. In nuclease-mediated gene editing, a double-strand DNA break (DSB) is created by a nuclease with a programmable target site ideally cutting the genome only at a single place close to the intended genomic alteration.

An early nuclease-based model system to study DSB repair in vivo used the Tet-Off or Tet-On controlled expression of the I-SceI endonuclease, which has an 18 bp recognition sequence that is not present in the mouse or human genome.

Nowadays, however, mainly nucleases with programmable target sites are used. Zinc finger nucleases (ZFNs) and transcription activator-like effector nucleases (TALENs) were the first widely used genome editing enzymes [4,5,6]. They rely on protein–DNA interaction, which means that a newly designed and constructed editing protein is required for each new genomic target site. This feature makes their generation both time- and resource-consuming [3]. These nucleases have been largely replaced by the Clustered Regularly Interspaced Short Palindromic Repeats (CRISPR)-associated nucleases (CRISPR/Cas) [7,8,9,10,11,12]. Here a guide RNA (gRNA) pairs with a Cas protein to form a complex that identifies a target 20 nt DNA sequence adjacent to a short protospacer adjacent motif (PAM). Among the various naturally evolved CRISPR-Cas systems, the type II DNA-targeting endonuclease Cas9 is most widely used for genome engineering [13]. Cas9 proteins isolated from different organisms use different PAM sites. Most widely applied is Cas9 of Streptococcus pyogenes (SpCas9), which creates blunt-ended DSBs and uses “NGG” as PAM. Cas12 creates 5 nt 5′-overhang DSB, and Cas12 from Francisella novicida requires “TTTN” as PAM sequence.

Following the binding to the DNA, the Cas protein cleaves the target site. If a template DNA carrying the corrected DNA sequence flanked by homologous DNA identical to DNA in the flanking regions of the DSB is provided, a homology-directed repair (HDR) can take place. By this mechanism, the defective DNA will be replaced by the corrected version, resulting in precise gene editing without any unwanted genetic alteration. HDR can induce any type of genetic change including point mutations, insertions, or deletions. It is the DNA sequence of the repair template that directs the genetic changes. Repair templates are single-stranded (ss) or double-stranded (ds) DNA, which employ different molecular repair mechanisms. Importantly, dsDNA templates are associated with an increased risk for toxicity and off-target integrations [14]. If no repair template is provided, the DSB will be closed by non-homologous end-joining (NHEJ). This process can introduce insertions and deletions of different lengths (indels), which often result in a frameshift when occurring within the coding region of a gene. HDR mainly takes place in the S/G2 phase of the cell cycle, while NHEJ shows no cell cycle dependence. For most cells, NHEJ is much more efficient than HDR.

For many clinical applications of gene editing, precise repair by HDR is the method of choice. However, imprecise, error-prone NHEJ may also be applicable in some cases. For example, many disease-causing mutations in Duchenne muscular dystrophy (DMD) can be treated by removal of one or several exons or by mutating an intron–exon border, causing skipping of the exon with a disease-causing genetic alteration [15]. The repaired dystrophin will be shorter than the wild-type version but will still be largely functional, resulting in therapeutical benefits. NHEJ-mediated DSB repair can be used to efficiently cause such deletions or mutations.

Another NHEJ-based method that can be used to replace defective genes with wild-type versions is homology-independent targeted integration (HITI) [16,17]. NHEJ can lead to the complete integration of a dsDNA fragment lacking homologous arms at the site of the CRISPR/Cas9-induced DSB. However, this integration will not be directed and might occur in the wrong orientation. In HITI, the end sequences of the dsDNA fragment will recreate recognition sites for the CRISPR/Cas9 used for introducing the genomic DSB when the integration occurs in the wrong orientation. Wrongly oriented fragments are therefore cut out again. When integrated into the correct orientation, the CRISPR/Cas9 recognition sites are not restored and the integration is stable. This trick results in a preferential integration in the correct orientation in an NHEJ-mediated repair process.

Base editing (BE) represents a novel approach that enables the generation of mutations by single-base modification without the need to induce DSBs, which prevents the occurrence of unwanted NHEJ-mediated repair [18]. For base editing, a nuclease-deficient Cas9 mutant is fused with a deaminase, which catalyzes the removal of an amino group from a nucleotide. Following DNA repair or replication, the deaminated nucleotide will be replaced by a nucleotide different from the original one. Different base editors have been developed that result in the substitution of C to T by Cytosine Base Editor (CBE), A to G by Adenine Base Editor (ABE), or C to G by C:G to G:C Base Editors (CGBEs). The deamination takes place at a certain distance from the genomic binding site directed by the gRNA and can stretch over several nucleotides. The applicability of BE is currently limited as not all types of base substitutions can be achieved, and it does not address gene defects that require insertions or deletions. Additionally, BE may modify all instances of a specific base within its action window, rather than just the base that should be corrected. On the other hand, BE does not require a repair template, which makes the transfer to the target cells easier.

Prime editing (PE) employs a modified Cas9 enzyme (nCas9) that nicks a single DNA strand, avoiding the creation of DSBs [19]. The nCas9 is fused with a reverse transcriptase (RT), which synthesizes DNA from an RNA template. Unlike conventional gRNA, PE uses a prime editing guide RNA (pegRNA), which includes an extension that encodes the desired genetic modification. The nCas9-RT fusion protein nicks the target DNA strand, allowing the RT to use the pegRNA as a template to extend the nicked strand with the new DNA sequence. This edited strand is then integrated into the genome through the cell’s native repair mechanisms, a process that can be enhanced by additionally nicking the non-edited strand [19]. Prime editing can introduce point mutations, and short insertions or deletions. Despite its potential, ongoing research is needed to fully explore and optimize the capabilities of PE, particularly in terms of efficiency, specificity, and the range of editable genomic contexts.

## 3. Gene Editing Reporters

In the majority of cases, reporters are utilized in a switch-on mode, meaning that they are expressed only in cells that have undergone successful gene editing. This approach minimizes background noise and eliminates the delay associated with the degradation of residual marker mRNA and proteins; this process is required in the switch-off mode and that can take several days.

The specific requirements for reporters in gene editing depend on the method of gene editing. For template-directed repair induced by DSBs, reporters need to be capable of detecting not only the desired HDR outcome but also any unintended NHEJ events.

Reporter genes with an insertion, deletion, or point mutation can be activated through HDR. Additionally, frameshift mutations are utilized to identify indels introduced through NHEJ. For BE applications, the reporter must be sensitive to single base-pair substitutions occurring within the activity window of the editor. Additionally, if there is potential for unwanted bystander mutations, the reporter system should be able to differentiate them from the intended mutation to accurately reflect the specificity of the BE process. PE can activate marker genes with point mutations or with such sufficiently short insertions or deletions.

The reporter gene is typically an enzyme that catalyzes a chemical reaction or a protein easily distinguishable from the background proteins. Three types of reporter genes have been used in the evaluation of in vivo gene editing: non-fluorescent reporters, luciferase reporters, and fluorescent reporters. Non-fluorescent reporters have been used for decades but are less popular now due to their generally low sensitivity. Luciferase reporters require an additional luciferin substrate for light emission in vivo, complicating their applications. Fluorescent reporter genes, for instance the GFP gene, are commonly used in gene editing reporter mice [20,21].

Fluorescence microscopy allows for the detection of these reporters within tissue sections at subcellular resolution. Autofluorescence, particularly in the green channel, may interfere with the detection of low levels of marker protein. The combination of fluorescent proteins with different absorption and emission spectra enables the simultaneous detection of two or three markers. To minimize “bleed-through” from spectral overlap, careful selection of absorption and emission filters is crucial. Moreover, fixation processes of tissues can significantly reduce the fluorescence intensity of marker proteins, while the thawing of unfixed cryosections might lead to the diffusion of fluorescent proteins from damaged cells [22]. Therefore, care should be taken to enable optimal detection of fluorescent marker proteins in tissue sections by fluorescence microscopy. In the case of fixation-caused loss of fluorescent signal or of low expression, marker proteins could be detected by immunofluorescent staining. The addition of a tag that restricts the marker to a specific subcellular localization such as the nucleus could facilitate detection. For single-cell analysis, fluorescence-activated cell sorting (FACS) is commonly used, especially in the gene editing of hematopoietic cells. While high and ubiquitous marker expression facilitates the analysis, it should be considered that fluorescent proteins are not biologically neutral and could have toxic effects or be the target of an immune response [23].

Reporters are exogenous genes that should be activatable strongly in all cells. They should be introduced into the mouse genome at a place where they can be highly expressed, but do not affect the expression of neighboring genes. Furthermore, their integration into the genome should not result in a phenotype by interfering with an endogenous gene at this genomic location. The Rosa 26 locus on chromosome 6 contains three exons that are spliced together but do not produce any protein. The transcription of the Rosa26 locus is ubiquitous and active across all cell types and developmental stages [24,25]. These features make the Rosa26 locus a preferred site for gene integration, often referred to as a “safe harbor” in mice. By integrating transgenes at the Rosa26 locus, researchers can ensure that the gene of interest is expressed in all cells of the organism.

## 4. Non-Fluorescent Reporter Mice

The lacZ gene is a widely used nonfluorescent reporter gene [21]. It encodes β-galactosidase, which facilitates the hydrolysis of various synthetic substrates. These substrates include chromogens like o-nitrophenol β-D-galactopyranoside (ONPG), 5-bromo-4-chloro-3-indolyl-β-D-galactopyranoside (X-gal), and 3,4-cyclohexenoesculetin-β-D-galactopyranoside (S-gal), producing distinct yellow, blue, and black products, respectively [26]. The resultant color change is readily detectable, allowing the lacZ reporter gene to enable sensitive and quantitative photometric analysis of cell lysates, but also in tissue sections.

Non-fluorescent reporters have been used for decades due to their stability and resistance to degradation, providing reliable data. However, they are generally less sensitive than other reporter systems and require additional reagents or substrates for the assay, complicating their application. The lacZ reporter has been used for investigating gene repair efficiency in vitro [27]. Furthermore, a lacZ reporter mouse (Table 1) for HDR has been made and was tested for repair with an ssDNA oligonucleotide, although in the absence of a nuclease-mediated DSB close to the mutation [28]. No repair was detected, but DSB induction by Cas9-gRNA will most likely lead to lacZ-positive cells detectable by staining of sections or tissue whole mounts.

## 5. Luciferase Reporter Mice

The luciferase gene is used as a marker for detection in live mice. Luciferase is an enzyme that generates bioluminescence and is widely used in biological research as a reporter gene. Originating from different sources, luciferase is divided into bacterial or eukaryotic types. One of the most commonly used variants is firefly luciferase, which, in the presence of magnesium ions (Mg^2+^) and oxygen (O_2_), catalyzes the ATP-dependent oxidative decarboxylation of luciferin, resulting in light emission at 526 nm [35]. The high sensitivity of this reaction and the direct correlation between the luciferase protein concentration and luminescence output make it an effective tool for real-time monitoring of gene expression.

After luciferin injection, a bioluminescent signal from luciferase-expressing cells can be detected in anesthetized animals [36,37]. This method facilitates whole-body analysis but lacks cellular resolution and detailed insights into the affected cells. It is particularly useful for longitudinal studies to track the fate of recombined cells.

Recent advancements have integrated luciferase with gene editing technologies. S. Y. Yu et al. [29] developed the LumA reporter mouse model (JAX #:038165) (Table 1), which can be used to evaluate the efficacy and safety of different genome editors, Lipid-Mediated Nanoparticle (LNP) formulations, and tissue-specific delivery systems. The luciferase gene in this model has a nonsense mutation at c.A1159T, resulting in a premature stop codon at amino acid 387 (R387X), rendering the luciferase nonfunctional before gene correction. This mutation can be efficiently corrected through A-to-G editing by SpCas9 ABE. By administering ABE mRNA and guide RNA to mice, they successfully detected luminescence through whole-body bioluminescence imaging, demonstrating its suitability for comparing the editing efficiency of different generations of base editors. Additionally, they assessed two FDA-approved lipid nanoparticles, ALC-0315 and MC3, and found that ALC-0315 had significantly higher delivery efficiency in the liver.

Luciferase has also been utilized to monitor NHEJ repair in gene editing. L. Amoasii et al. [30] ligated a luciferase reporter gene after the dystrophin gene in normal mice, and both genes were expressed (WT-Dmd-Luc). To study Duchenne muscular dystrophy (DMD), they knocked out exon 50 of the dystrophin gene in the WT-Dmd-Luc mouse, resulting in a frameshift mutation starting from exon 51, including the luciferase gene, creating the ΔEx50-Dmd-Luc DMD disease mouse model (Table 1). A CRISPR/Cas9 with an sgRNA targeting the exon 51 splice acceptor site was administrated to restore the dystrophin gene. By skipping exon 51 through NHEJ, the dystrophin gene and luciferase gene were shifted to the frame, and the correction efficiency could be evaluated by luciferase expression. Their findings indicated that four weeks post-treatment with AAV9 vectors delivering Cas9 and guide RNA, the bioluminescence intensity in the ΔEx50-Dmd-Luc mice closely matched that of the WT-Dmd-Luc mice. This innovative use underscores luciferase’s potential for tracking and quantifying NHEJ in testing of therapeutic gene editing. The luciferase reporter system is a highly sensitive and reliable tool, capable of detecting low levels of gene expression and providing accurate quantification across varying expression levels. However, it requires additional luciferin as the substrate for light emission, and the luminescence signal decays relatively quickly, requiring rapid measurements for accurate quantification.

## 6. Fluorescent Reporter Mice

The green fluorescent protein (GFP), originally isolated from the jellyfish Aequorea victoria, is the most well-known fluorescent protein [38,39,40]. Over time, additional color variants such as blue, yellow, and red fluorescent proteins have been developed [26].

Fluorescent reporters are easy to detect by FACS in a single-cell suspension and are of excellent value for testing targeting and repair efficiency in cell lines. In live reporter mice, the fluorescence signal will only be detectable in the top cell layers of the skin and require shaving of the fur, feeding with low fluorescent food, and preferably also an albino background to reduce background fluorescence [41,42]. However, isolated primary cells from the hematopoietic system but also from solid organs could be easily investigated by FACS and differentiated by cell surface markers for specific cell types and stem cells. While fluorescent proteins can be detected by fluorescence microscopy on sections, it needs to be considered that standard PFA fixation will decrease the fluorescence signal, making immunofluorescent or immunohistochemical staining of the fluorescent reporters sometimes the preferable detection method.

**Figure 3:**
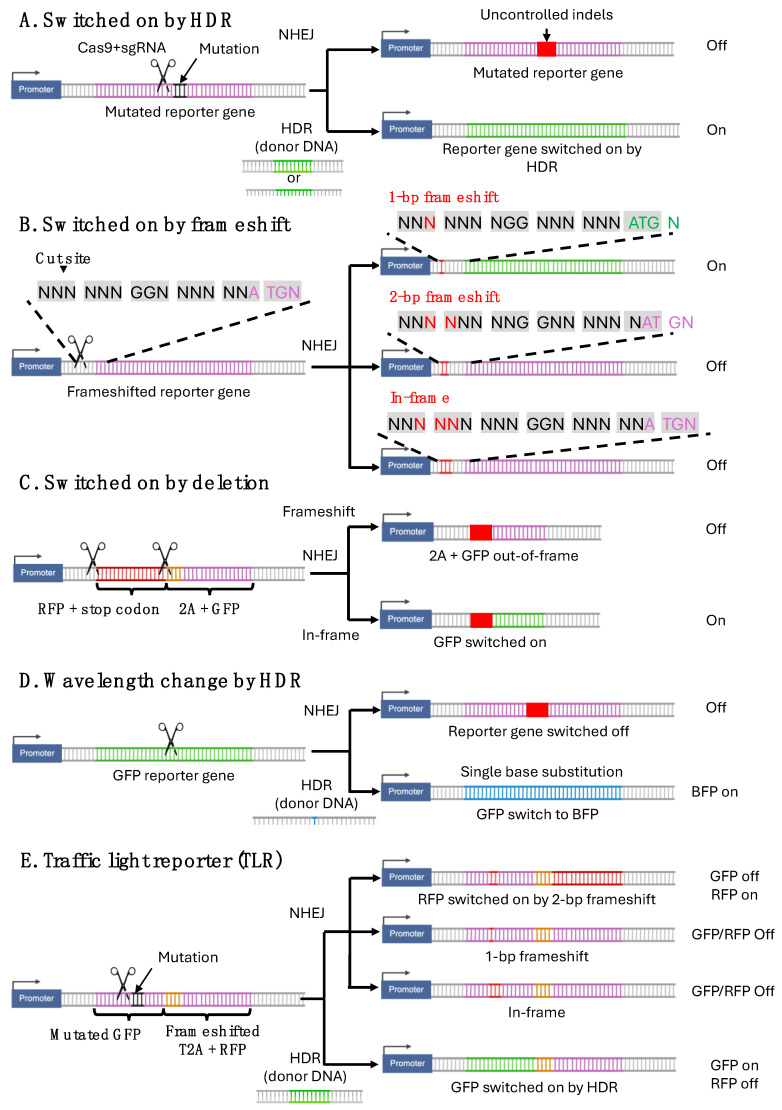
Fluorescent reporter systems to detect gene editing outcomes. Purple base pairs: non-fluorescent reporter. Red, green, or blue base pairs: fluorescent reporter. Black base pairs: reporter inactivating mutation. Orange base pairs: 2A sequence allowing the production of two proteins from a single transcript, by ribosomal skipping. Red box: Insertion or deletion (Indel). Gray boxes: Codons. Scissors: Cut site for Cas9/sgRNA.

Different types of fluorescent reporters for gene editing have been established (Figure 3). Repurposed Cre reporter mice allowed the detection of NHEJ repair by fluorescent reporters. The Cre recombinase is a 38 kDa protein that recognizes and mediates site-specific recombination between 34 bp sequences referred to as loxP (locus of crossover [x] in P1 bacteriophage) site [43]. This Cre-loxP system allows precise temporal and spatial control over gene knock-out in mice [44]. By excising genes flanked by loxP sites (also known as “floxed” genes) through intrachromosomal recombination, Cre recombinase generates conditional knock-outs [31]. By removing a stop cassette between promoter and gene of interest, the Cre-loxP system can also be used to conditionally switch on gene expression. Fluorescent reporter mice with a floxed stop cassette in front of a fluorescent reporter were made to characterize the efficiency and tissue specificity of transgenic Cre mice. However, these reporter mice can also be used to test CRISPR gene editing in vivo.

The global double-fluorescent Cre reporter mouse, created by M. D. Muzumdar et al. [41], includes a floxed membrane-targeted tdTomato (mT) and a membrane-targeted EGFP (mG) (R26 mT/mG) (JAX #:007676) (Table 1). Initially, only mT is expressed. In the presence of Cre recombinase, mT is excised and mG is expressed. Instead of Cre, Alapati et al. used CRISPR gene editing and NEHJ-dependent repair to remove mT and activate mG in the lungs of fetuses of the R26 mT/mG reporter mice [45]. Adenovirus (Ad) vectors containing SpyCas9 and a gRNA targeting the loxP sites flanking the mT gene were delivered via the intra-amniotic pathway. The amniotic fluid, containing the Ad vectors, enters the lungs through the fetus’s inhalation during respiratory movements, leading to efficient pulmonary gene editing. Fluorescent stereomicroscopy of the lungs revealed a strong change from red to green fluorescence, and FACS analysis showed mG expression in epithelial cells (18%), endothelial cells (0.2%), and mesenchymal cells (1.8%) after editing. For analysis of sections, immunofluorescence microscopy was used, suggesting that direct detection of fluorescence of mT and mG was not efficient enough.

More recently, an EGFP-based single reporter mouse for the detection of HDR was generated by CRISPR-mediated mutations in the EGFP coding sequence of a transgenic mouse with a single copy of EGFP under the control of the ubiquitously active CAG promoter [32] (Table 1). Mice with mutations that abrogated the fluorescence of EGFP can be used as reporter mice for precise gene editing by HDR. Unexpectedly, one of the mouse lines generated showed restoration of EGFP expression by an NHEJ-mediated in-frame deletion, indicating that all reporter mice should be tested for expression of the HDR marker after a DSB repair in the absence of a repair template to exclude NHEJ-dependent activation of the HDR reporter. This single reporter system was used to test HDR following zygote injection, intra-oviductal injection, and in vivo electroporation of the entire oviducts. This reporter mouse has been used to optimize the delivery of ribonucleoproteins (RNPs) to the uterine epithelium for the treatment of endometrial carcinoma (EMC) [46].

To distinguish between DSB repair by NHEJ and HDR through fluorescent reporters, Certo et al. [47] developed the traffic light reporter (TLR) system, which detects HDR and NHEJ repair of a DSB by green and red fluorescence, respectively. This system includes a nonfunctional mutated EGFP gene with an embedded cleavage site for the nuclease I-SceI, followed by a 2 bp reading frameshifted self-cleaving 2A peptide and a red fluorescent mCherry gene. The 2A sequence allows the production of two separate proteins from a single mRNA by ribosomal skipping. The reporter is designed in a switch-on mode since initially it does not express any fluorescent signal. If a DSB introduced by expression of I-SceI is repaired in the presence of a repair template through the HDR pathway, the EGFP sequence will be reconstituted, resulting in cells fluorescing green. If the break is repaired via mutagenic NHEJ introducing indels, the EGFP function will not be restored. However, an indel frame shift may be introduced, which brings the T2A and mCherry sequences in-frame to produce red fluorescent cells. Using HEK293 cells stably expressing this double fluorescence reporter, researchers can quantify HDR efficiency and 2 bp frameshift mutations through flow cytometry. The TLR system offers the benefit of straightforward monitoring and quantification of intracellular DSB repair efficiency.

This TLR approach was later applied to also monitor CRISPR genome editing. Following the same TLR reporter concept, V. T. Chu, et al. [33] replaced codons 117–152 of the Venus fluorescent protein with target sequences from the mouse Rosa26 and Rab38. Introducing a DSB into the replacement part by Cas9-gRNA and providing a dsDNA repair template, they could restore a functional Venus protein. NHEJ-dependent indels rescuing the frameshift mutation allowed the expression of a TagRFP red fluorescence. Both Venus and TagRFP have a high brightness, facilitating detection. Using this novel TLR cell line, they successfully quantified the in vitro efficiency of HDR and 2 bp frameshift NHEJ by flow cytometry and evaluated the effectiveness of various shRNAs and small molecule inhibitors in suppressing NHEJ. Mice with the TLR inserted into the Rosa26 locus have been generated and are available at JAX (#:034033) (Table 1).

S. Iyer et al. [34] modified the Certo TLR vector by introducing PAM sequences for multiple Cas9 and Cas12 orthologs into the non-functional GFP transcript. This modification allows various Cas proteins to introduce DSBs at the same location. The redesigned TLR-MCV1 was integrated into HEK293T and K562 cells, enabling the evaluation of HDR/NHEJ gene editing efficiency across different cell types, Cas9 proteins (SpyCas9 and AspCas12a), and donor templates.

The Watts group [48] integrated this TLR-MCV1 into the Rosa26 locus, a “safe harbor” in the mouse genome, to create the R26-TLR-MCV1 mouse (JAX #:038717) (Table 1). Using FACS analysis of primary MEFs, they monitored nuclease-mediated editing by HDR or NHEJ in vitro. In vivo, they only tested NHEJ following AAV-mediated transduction with Cas9-sgRNA. Repaired mCherry was detected by immunohistochemical staining of sections of the liver, heart, and lung. Furthermore, they used PE to repair the mCherry or the EGFP reporter in vivo, demonstrating that this reporter mouse can also be used to test methods for delivering PE. Their results suggest that the direct application of Cas9-gRNA ribonucleoprotein complexes is more efficient than a vector-based system expressing both Cas9 and gRNA for in vivo gene editing.

As an alternative to the double reporter approach, Glaser et al. reported a system where HDR directs a switch of the emission wavelength of a fluorescent protein [49]. They developed a new reporter system by exploiting the fact that a single base substitution (196T>C) in EGFP shifts its absorption and emission spectra toward blue. They introduced a stably expressed EGFP gene into K562 and HEK293T cells, which was subsequently edited using CRISPR/Cas9 and a short ssDNA repair template. HDR and NHEJ were quantified by measuring the blue fluorescence signal and the loss of green fluorescence, respectively. For the potential in vivo application of this system, it should be considered that it will be probably very difficult to distinguish the highly GFP and BFP proteins by immunofluorescence if the direct detection of GFP and BFP fluorescence in the PFA fixed section might not result in a sufficient signal. Due to the stability of the fluorescent proteins, it was required to wait before conducting analysis of the edited cells until the EGFP protein of the NHEJ repaired cells was not detectable anymore. This system has the advantage that it will recognize all frameshifting indels and not only a fraction of them, such as in the TLR systems described above. No reporter mouse has been described with this system.

## 7. Conclusions

The therapy of rare diseases caused by gene defects is an important unmet clinical problem. Gene editing therapy repairing the defect is an exciting therapeutic option with curative potential. However, the poor efficiency of gene editing, risks with respect to patient safety, and high costs are major obstacles to be overcome. Optimizing the efficiency of gene editing therapy will benefit patients suffering from many different rare diseases. Here, reporter mice are crucial tools for quick in vivo testing of novel tools for CRISPR-based genetic editing and for methods of transferring these tools to the target cells. They can also give an indication of safety issues and long-term therapeutic effects. Finally, they can be used to isolate tissue-specific stem cells for testing of ex vivo gene therapy approaches. Several reporter mice have been established, but there seems to be room for improvement. To our mind, the ideal reporter mouse should allow the distinction of HDR and NHEJ by FACS or fluorescence microscopy. Importantly for NHEJ-mediated repair, all out-of-frame mutations should be detected. Since dsDNA and ssDNA repair templates are employing different molecular mechanisms for precise genome editing of DSB, the reporter system should be able to test both methods. If the reporter could also be used for PE and BE using the same sgRNA, this would facilitate the comparison of DSB repair by HDR with PE or BE. Such reporter mice can be made with the existing toolbox of reporter systems and would have the potential to become a standard tool for developing and benchmarking novel technologies for precise gene editing of rare diseases.

## Figures and Tables

**Table 1:** Overview of reporter mice for analysis of gene editing efficiency.

	Name	Reporter	Detected Repair Mechanisms	JAX Number	References
1	Gtrosa26^tm1-Col^ mouse	LacZ	HDR	-	[28]
2	LumA mouse	Luc	HDR, ABE	38165	[29]
3	ΔEx50-Dmd-Luc mouse	Luc	NHEJ	-	[30]
4	ROSA^mT/mG^ mouse	tdTomato, EGFP	NHEJ	007676	[31]
5	ΔEGFP transgenic (Tg) mouse	EGFP	HDR	-	[32]
6	R26-GFP_KI-TLR2 mouse	Traffic light reporter	HDR, NHEJ (+2 frameshift)	34033	[33]
7	R26-TLR-MCV1 mouse	Traffic light reporter	HDR, NHEJ (+2 frameshift), PE	038717	[34]
